# Airline transportation and arrival time of international disease spread: A case study of Covid-19

**DOI:** 10.1371/journal.pone.0256398

**Published:** 2021-08-19

**Authors:** Pei-Fen Kuo, Chui-Sheng Chiu

**Affiliations:** Department of Geomatics, National Cheng Kung University, Tainan, Taiwan; Ningbo University, CHINA

## Abstract

In this era of globalization, airline transportation has greatly increased international trade and travel within the World Airport Network (WAN). Unfortunately, this convenience has expanded the scope of infectious disease spread from a local to a worldwide occurrence. Thus, scholars have proposed several methods to measure the distances between airports and define the relationship between the distances and arrival times of infectious diseases in various countries. However, such studies suffer from the following limitations. (1) Only traditional statistical methods or graphical representations were utilized to show that the effective distance performed better than the geographical distance technique. Researchers seldom use the survival model to quantify the actual differences among arrival times via various distance methods. (2) Although scholars have found that most diseases tend to spread via the random walk rather than the shortest path method, this hypothesis may no longer be true because the network has been severally altered due to recent COVID-related travel reductions. Therefore, we used 2017 IATA (International Air Transport Association) to establish an airline network via various chosen path strategies (random walk and shortest path). Then, we employed these two networks to quantify each model’s predictive performance in order to estimate the importation probability function of COVID-19 into various countries. The effective distance model was found to more accurately predict arrival dates of COVID-19 than the geographical distance model. However, if pre-Covid airline data is included, the path of disease spread might not follow the random walk theory due to recent flight suspensions and travel restrictions during the epidemic. Lastly, when testing effective distance, the inverse distance survival model and the Cox model yielded very similar importation risk estimates. The results can help authorities design more effective international epidemic prevention and control strategies.

## 1 Introduction

The expansion of global business, recreational travel and the connection of regional economies have led to an increased demand for international travel. Thus, the airlines have become the most vital mode of transportation due to their speed, worldwide range and reliability. However, the potential threat that accompanies these major strides is that epidemics also spread more rapidly, causing global public health crises and the necessity for epidemic prevention [1, 2]. According to epidemiologists, infectious diseases are spread through contact in close quarters [3]. In earlier studies, some researchers predicted that risk of disease spread was based on the geographical distance of the airport connection network (World Airport Network (WAN)) of various countries [2, 4]. For example, Lemey used the stochastic Monte Carlo method to separate flight routes into various airport zones in order to explore the correlation of geographical distance and the transmission of H3N2 [5]. Similarly, the ranges of some mosquito borne diseases were found to be affected by geographical distance, such as dengue [6] and malaria [7]. However, this element ignores the major impact of modern transportation systems, particularly aviation [1, 8–10]. For example, although two airports may be very far away from each other, they would still experience heavy interaction because of busy flight schedules, which would accelerate epidemic spread over long distances. To solve this puzzle, Brockmann proposed the effective distance method, which is based on traffic flow, to predict global disease spread [11]. For example, although the Narita Airport and the Haneda Airport in Tokyo, Japan have a relatively close geographical distance (≈71 km), their effective distance is almost infinite because there are no regular flights between them, which makes it virtually impossible for COVID-19 to be transferred from these two airports by passenger plane. This new method allows researchers to convert airline passenger volume into a metric form that can be applied in epidemiological studies to predict the arrival dates of various types of diseases. For example, Lin used the effective distance of land traffic volume to estimate the regional transmission risk of COVID-19 [12]. These results showed that cities with higher connection density have more rapid disease spread (COVID-19). Additionally, Brockmann found effective distance to be highly correlated with the arrival time of SARS and H1N1 [11]. This method has also been used to estimate the risk of other infectious diseases, including Zika [13], influenza [14], and MERS [15].

In other words, graphical representations or traditional statistical methods were used in most previous studies to determine if effective distance can predict disease arrival time better/more accurately than geographical distance [5, 11, 16]. In addition, scholars have applied effective distance to establish transition probability in order to predict disease risk, possible case numbers, or arrival times [12, 13, 17]. However, none have incorporated different types of distance among airports (effective distance and geographical distance) into the survival model to predict arrival times and to compare the accuracy of these two estimators with the actual disease arrival dates in each country. Thus, by using a survival model and real data (IATA air traffic volume and real-world Covid-19 arrival data), we were able to measure the true/actual error rate of arrival time estimators and determine the difference between geographical distance and effective distance estimators. In addition to utilizing different types of distance as predictive variables (geographic distance and effective distance), the contribution of this study also includes the incorporation of two path selection strategies (shortest path and random walk) into two different survival models (the Cox model and the inverse effective distance model).

The course of disease spread is another critical research topic [10, 18]. In the past, many scholars assumed that a disease would spread via the shortest path due to having the shortest transmission time [5, 13]. However, it is now known that diseases may be transmitted via all possible paths rather than simply the shortest [5]. In order to test the accuracy of the random walk theory, Gautreau developed a probability function of disease spread via multiple paths, which inspired Brockmann’s concept of effective distance [10]. Almost ten years later, Iannelli utilized Gautreau’s findings to calculate the effective distance via the random walk and compared the results to those of the effective distance via the shortest [18]. While the shortest path walk involves a transmission route of the least length, the random walk path theory hypothesizes that the disease follows one path from all other possible paths based on probability.

Survival models are often used to predict the time an event occurred or will occur. The main reason is that the survival times are usually highly skewed, which causes the normality assumption to be violated. Thus, use of linear regression was inappropriate in this case. For example, the higher survival rates in some countries (that persist to this day and the fact that these countries have not had any newly imported Covid-19 cases) have caused our arrival time data to be impacted by an atypical skewed distribution and censoring bias. With its probability function, the survival model can solve these problems by estimating the probability that an event will occur at each time t, and the degree of danger that disease arrival presents. In this study we used the most straightforward and common methods (the inverse effective distance and the Cox model), to estimate the probability of arrival time. The Cox model includes the hazard function which can be used to estimate the impacts of the explanatory variables, such as effective distance in this study [19]. Although few scholars have used the survival model to predict disease arrival time, none have compared the prediction performance of the inverse distance model to the Cox model for each country. Lastly, few researchers have used historical airline datasets to examine effective distance and path selection. However, it must be noted that due to the recent decrease in air traffic volume because of travel restrictions, the effective distance estimator and the random walk path may not be the most effective techniques for analysis of disease spread.

To account for these limitations, we compared the performance of various distances and path selection prediction models. (1) The effective distance was calculated based on the IATA flight database, and the geographic distances was calculated among the WAN airports. (2) We then utilized the different path approaches (random walk and shortest path) to establish the airport network. (3) Then, two survival models were employed to quantify the prediction performance for importation risk.

## 2 Materials and methods

### 2.1 Study data

The goal of the current study was to define how passenger airline travel has influenced the international spread of COVID-19. Our hypotheses are briefly stated below:

1. Effective distance will have a significantly higher positive correlation with disease arrival time in various counties than will geographical distance.
2. The random walk effective distance will have a higher positive correlation with arrival time than will the shortest path.
3. The effective distance can be included in the survival model to estimate the risk of spreading the disease to various countries.

Therefore, the study data included both a disease dataset and an airline dataset. In order to limit this study to cases connected with air transportation, we focused exclusively on the first-confirmed incidences reported in various countries and searched the associated airports to find those who were first infected.

**Fig 1:**
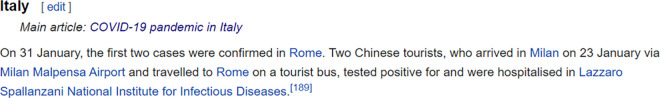
Screenshot of the “COVID-19 pandemic in Europe” Wiki page.

Since COVID-19 is a progressive pandemic, up-to-date data was continuously collected from news websites. The disease arrival time in each country was based on information published on Wikipedia, which not only provides the most multi-faceted and current information but it also is overseen by a volunteer editor. Unlike other official data sources, such as The Johns Hopkins Coronavirus Resource Center and The Program for Monitoring Emerging Diseases (Promed), Wikipedia includes more recent and detailed information about the spread of this deadly disease at (https://en.wikipedia.org/wiki/COVID-19_pandemic_by_country_and_territory). We collected unstructured data manually and extracted the dates of the first cases reported/first confirmed cases. For example, Fig 1 shows a screenshot from a Wiki page of COVID- 19 spread in Italy in late January. From this first-case information, the arrival time (January 31) and original airport (Milan Malpensa Airport) can be determined.

Due to the fact that Wikipedia’s data sources come from volunteers with varying levels of expertise, we also utilized Promed (Program for Monitoring Emerging Diseases) to double check this dataset. For this study, we downloaded 160 Covid-19 reports from the Promed system during our study period (from 12th January to 21st April, 2020), and searched the reported time of first confirmed case via country name. We utilized this arrival date as a reference point to doublecheck Wiki’s dataset. For example, according to Wiki news, the first confirmed Covid-19 case in Italy was reported on 30th January. We compared this with the Promed report, which corroborated this information (shown as below). Based on this method, there are 69 countries (34% of all countries with Covid-19), and their information was verified/double checked with Promed.) It must be noted that Promed lacked detailed data on 109 countries.

The air transportation data in this study, provided by the International Air Transport Association (IATA). IATA, included only international airlines and passenger flights rather than code-sharing airlines, cargo flights, or the transfer airports. After calculating the number of flights in service during the study period, we found that IATA had many flights scheduled from Málaga Airport (AGP) to Bucharest Henri Coandă International Airport (OTP), as shown in Table 1. For example, after calculating the service days, it became clear that flight number 0B126 was in service once per day on Tuesdays, Thursdays and Sundays from January 3, (the scheduled effective date), to March 23, 2017, the (scheduled discontinued date). Later in the year, flight number 0B126 would be in service once per day on Thursdays, Saturday, and Sundays from March 26, the scheduled effective date, to October 28, 2017, the scheduled discontinued date, etc.

**Table 1:** An example of IATA flight data.

Flight Number	Effective	Discontinued	Day1	Day2	Day3	Day4	Day5	Day6	Day7	Departure	Arrival	Service
	Date	Date								Airport	Airport	days
0B 126^a^	03/01/2017	23/03/2017	0	1	0	1	0	0	1	AGP	OTP	35
0B 126^a^	26/03/2017	28/10/2017	0	0	0	1	0	1	1	AGP	OTP	93
0B 126^a^	27/03/2017	24/10/2017	1	1	0	0	0	0	0	AGP	OTP	62
0B 126^a^	29/10/2017	24/03/2018	0	0	0	1	0	1	1	AGP	OTP	63
0B 126^a^	30/10/2017	20/03/2018	1	1	0	0	0	0	0	AGP	OTP	42
W6 3190^b^	28/03/2017	28/10/2017	0	1	0	0	0	1	0	AGP	OTP	62
W6 3190^b^	31/10/2017	24/03/2018	0	1	0	0	0	1	0	AGP	OTP	42

^a^ 0B: Blue Air Aviation.

^b^ W: Wizz Air Hungary Ltd.

**Fig 2:**
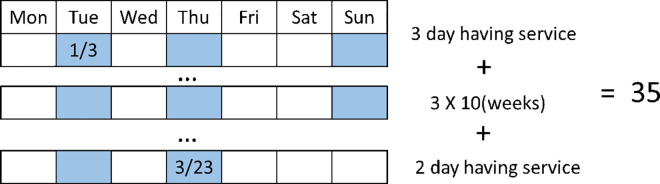
An example of flight number calculation.

During the first period, there were 35 flights in service for 10 weeks. As shown in Fig 2, there were two in service during the last week of the first period. The first period flight consisted of 11 weeks which had three service days, so this flight had a total of 35 service days (3×11+2 = 35). When all flights shown in Table 2 were combined, # 0B126 had a total of 295 (= 35+93+62+63+42) service flights. When all service flights from AGP to OTP (such as 0B12 and W6 3190), were added up, there were 399 in this O-D pair (AGP-OTP) from the original (AGP) to the destination airport (OTP). The corresponding fraction ratio was calculated from flights in service in this O-D pair (AGP-OTP) divided by all service flights from the original airport (AGP) (= 71,980). Thus, the effective distance of the O-D pair (AGP-OTP) was equal to one minus the fraction ratio, which was 6.195 (≈ 1-log ($\frac{399}{71980}$)).

**Table 2:** Comparison table of Wiki news and Promed report.

resource	Successful verified/match	Fail to verify/unmatched
Wiki News	**On 30 January, the first two cases** were confirmed in Rome. Two Chinese tourists,…	The **first two confirmed cases** in Vietnam were admitted to Cho Ray Hospital, Ho Chi Minh City on **23 January 2020**
Promed report	On Thursday **[30 January 2020], Italy reported the 1st confirmed cases** of infection in two Chinese tourists, currently hospitalized, in isolation, at the Lazzaro Spallanzani Reference Center in Rome.	There have also been 4 suspected cases in Taiwan,2 in Vietnam.
		**(Promed just included the number of cases in Vietnam but did not mention the reported time of the 1** ^**st**^ **confirmed case.)**

**Fig 3:**
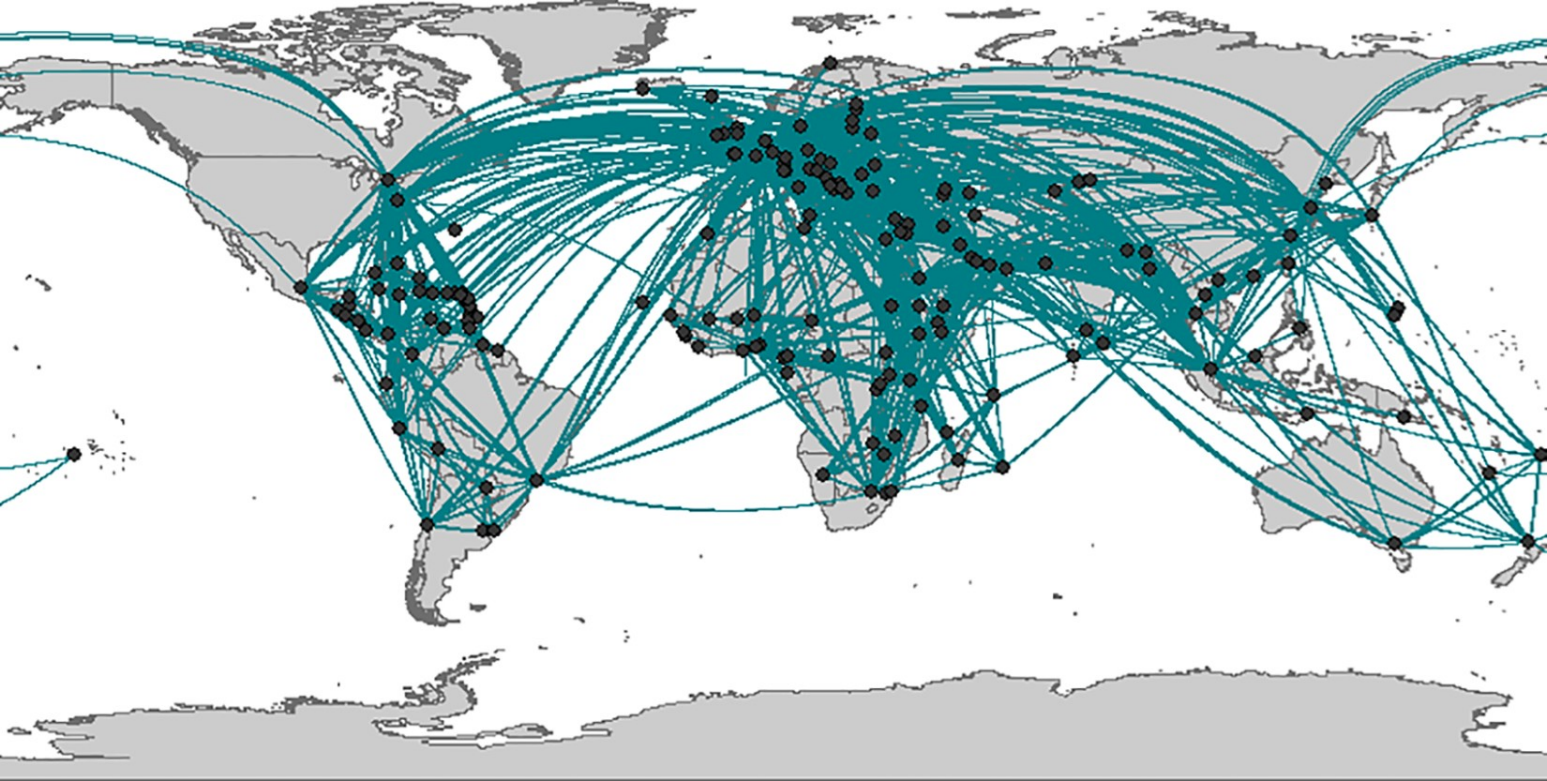
The airport network of the COVID-19 arrival airports.

During the one-year study period (2017), 1,312 airports and 14,707,367 flights were included in the IATA dataset. The O-D matrix in our study was established from arrival and departure airport codes. The airline data and the frequency of flights during this year were summarized to generate an O-D pair dataset, which included the annual flight frequency from each O-D line (origin airport to destination airport). By combining the COVID-19 cases and IATA airport datasets, the originating importation airport from the IATA database was determined based on the importation information. Fig 3 displays the flights between COVID-19 arrival airports used in this study. The 178 points represent the airports which imported confirmed COVID-19 cases, and the lines represent the flights between these airports. It should be noted that each line has a uniform thickness, simply represents the Covid-19 airport network (which imported the first Covid-19 case). Because some lines overlap with each other near the hub/large airport, they appear to have different thicknesses.

### 2.2 Effective distance

The effective distance is equal to the arrival time multiplied by the effective speed. Brockmann [11] developed this approach to estimate the arrival times in various countries via the effective distance, (which is calculated only by the percentage of passengers) divided by the effective speed (which is measured by epidemiological parameters, such as the transmission rate) [11], which is shown below:

$$d_{ij}^{eff}=1-\ln\frac{w_{ij}}{\sum_{j}w}$$ (1)

**Fig 4:**
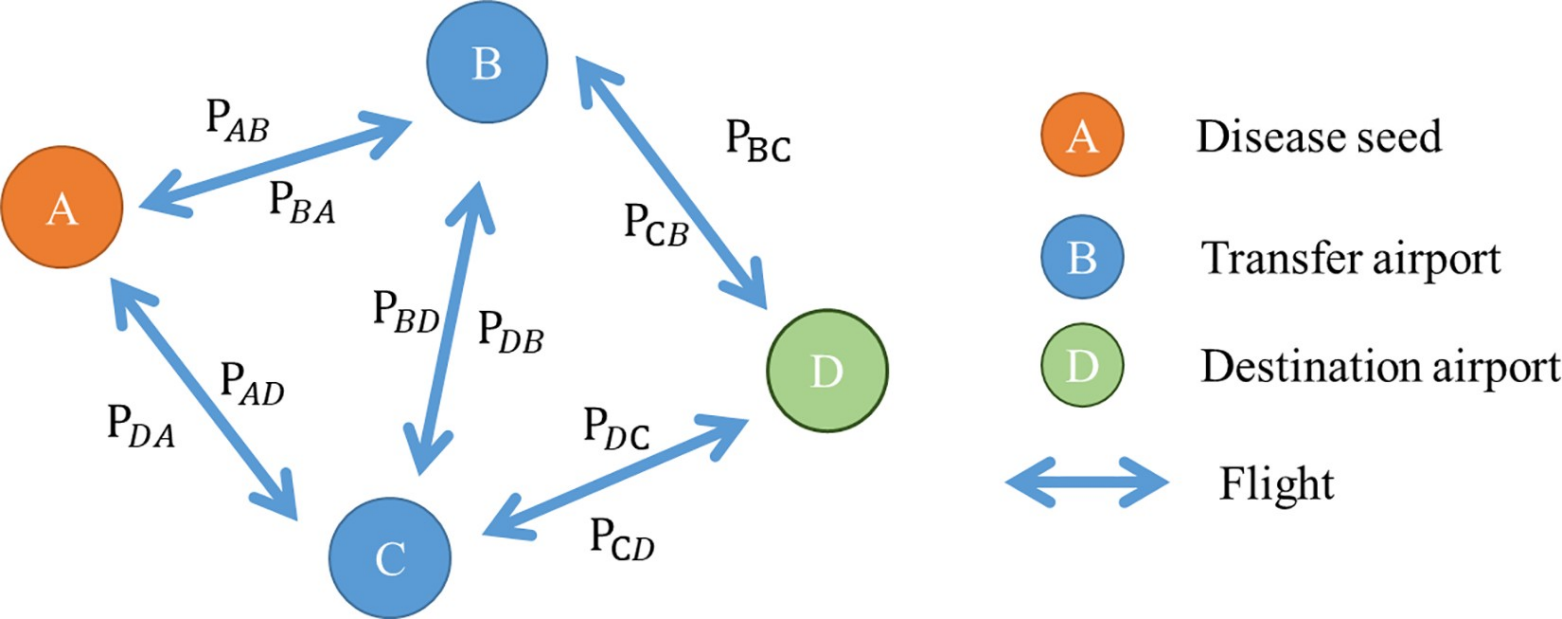
An example of an airport network.

As seen in Eq 1, ∑_***j***_***w*** is the total passenger flow form airport j. The effective distance from airport i to airport j ($d_{ij}^{eff}$) is determined by the percentage of passengers P ($\frac{w_{ij}}{\sum_{j}w}$). For example, in Fig 4, which shows an airport network, the links between airports is the flight, and the percentage of passengers from airport i to j is P_ij_ (equal to $\frac{w_{ij}}{\sum_{j}w}$). Thus, the effective distance between airports can be calculated.

### 2.3 Selection strategy of different paths

In Fig 4, airport A is the original airport (disease seed), B and C are the transfer airports, and D is the destination airport. We assumed that there is no direct flight between airport A (disease seed) and airport D; therefore, the infectious disease can only be transmitted to airport D by airports B or C. Previous researchers used the shortest path and random walk methods to determine the actual transmission path of disease spread, as discussed in the section below.

#### 2.3.1 Shortest path walk

In order to estimate risk at airports that are not directly linked to the disease seed airport, Brockmann used the shortest path technique to define the potential route of disease spread [11]. For this method, the path with the minimum total effective distance was selected as the most likely route for disease spread.


$$D_{ij}^{SP}=\underset{\Gamma_{\mathrm{ij}}}{\min}\sum_{{(k,l)\;\epsilon \;\Gamma }_{ij}}d_{kl}^{eff}$$ (2)

In Eq 2, Γ_ij_ represents all possible paths from airport i to airport j, in which all nodes cannot pass more than once. A path is composed of consecutive links (k,l), from i to j, and the minimum function is calculated via the minimum total length of effective distance. Although Brockmann found a high correlation between the shortest path walk and arrival time at airports, use of the former might cause researchers to overlook the probability of disease spread by different paths, resulting in overestimation of the arrival time [10, 20].

#### 2.3.2 Random walk path

Due to the abovementioned limitations, several scholars, such as Iannelli [18], later used the random walk theory to simulate how infectious diseases have spread via all possible paths within a transportation network. For example, as shown in Fig 4, there are four airports which are linked via flight patterns. In this scenario, airport A is the disease seed and we must determine the arrival time of the disease at airport D. While the shortest path walk is utilized to find the route with the least total effective distance (A to B and D), the random walk method is used to analyze all possible paths, noting that the paths may pass through one node more than once. This method is used to calculate the probability of disease spread by each path. Using the definition of effective distance described above, Gautreau determined that the arrival probability function of multiple paths can be calculated by utilizing Eq (3, in which $D_{ij}^{mp}$ is the multiple path effective distance, defined as the time at which the disease began to be transmitted via multiple paths [10]. Following this equation, the multiple paths can be determined by summarizing the exponentials of the effective distance of all possible paths.


$$e^{-D_{ij}^{mp}}=\sum_{path{\;\epsilon \;\Gamma }_{ij}}e^{-D_{path}^{eff}}$$ (3)

$$D_{ij}^{mp}=-\ln\left[\sum_{path\;{\epsilon \;\Gamma }_{ij}}\left(e^{-n_{path}}\times \prod_{(k,l)\;\epsilon \;path}P_{kl}\right)\right]$$

In Eq 3, $D_{path1}^{eff}$ represents the total effective distance via several paths, and *n*_*path*_ is the length of these “paths”; To simplify the effective distances of the multiple paths, Iannelli established the *F*_*ij*_(*n*) function to summarize the percentage of paths a passenger could take, which are included in Γ_ij_, with the same length n [18]. Thus, Eq (3 can be rewritten as Eq 4, as shown below.


$$D_{ij}^{mp}=-\ln\left[\sum_{n=1}^{n_{max}}e^{-n}F_{ij}(n)\right]$$ (4)

$$F_{ij}(n)=\sum_{|path|=n}\left[\prod_{(k,l)\;\epsilon \;path}P_{kl}\right]$$ (5)

In Eq 4, *n*_*max*_ symbolizes the maximum path length. Iannelli et.al 2017combined multiple path effective distance and random walk effective distance to account for all possible paths and the fact that these paths may pass through a node more than once [18]. These researchers replaced *F*_*ij*_(*n*) with an *H*_*ij*_(n) function that allows flights to pass through the paths more than once. Because every node can be reached several times and the maximum length of the path is infinite, a power series was used to simplify the formula into Eq 6:

$$D_{ij}^{RW}=-{\ln\left\{{\left[eI(j|j)-P(j|j)\right]}^{-1}p(j)\right\}}_{i}$$ (6)

$$=-\ln\left[\sum_{n=1}^{\infty }e^{-n}H_{ij}(n)\right]\;(∵power\;series\frac{1}{1-x}=\sum_{i}^{\infty }x^{i})$$

The H_ij_(*n*) function is part of Eq (6) as the nth power matrix of the percentage of passenger *s* P(j|j)H_ij_(*n*) = [*P*(*j*|*j*)^*n*^]_*ij*_, P(j|j) represents the percentage of the passenger matrix P that removes the jth row and jth column.), which means that the path was unable to reach the destination airport. This function is equal to the sum of the probability of disease transmission via the i to j path with “n” length nodes. Following the example shown in Fig 3, the infectious disease may spread from A→B→C or from A→D→C. On the other hand, because the disease may reach a node more than once [16], there may be many different transmission paths, e.g. A→D→A→B→C or A→B→D→B→C.

**Fig 5:**
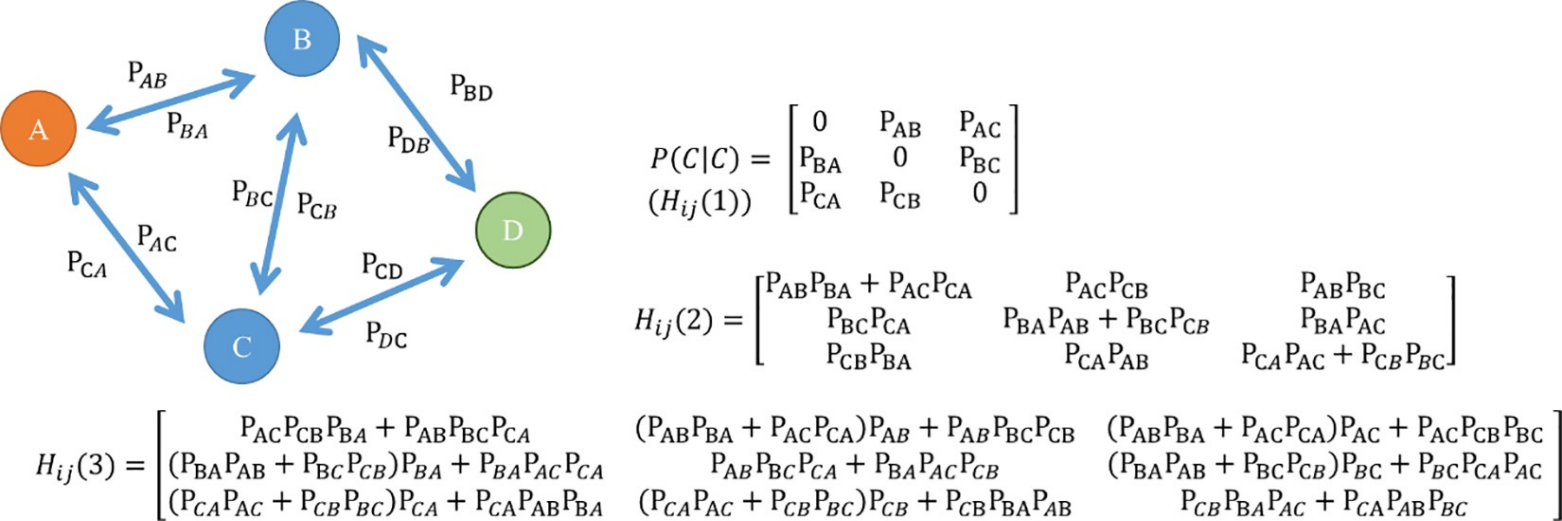
The calculation of H_ij_(*n*).

For more details, Fig 5 shows an example of an H_ij_ matrix. The percentage of passengers of H_ij_(1) is P(j|j), which means that the disease only comes into contact with one airport on its way to the destination airport and includes the percentage of passengers (P_AB_, P_AC_, P_BA_, P_BC_, P_CA_, P_CB_). Therefore, H_ij_(2) is the percentage of passengers via two nodes. For example, if those from A to B are equal to P_AD_ multiplied by P_DB_. (H_AB_(2) = P_AD_P_DB_), then, H_ij_(3), the path from A to B would also include path A, A→D→A→B.

### 2.4 The survival model

**Fig 6:**
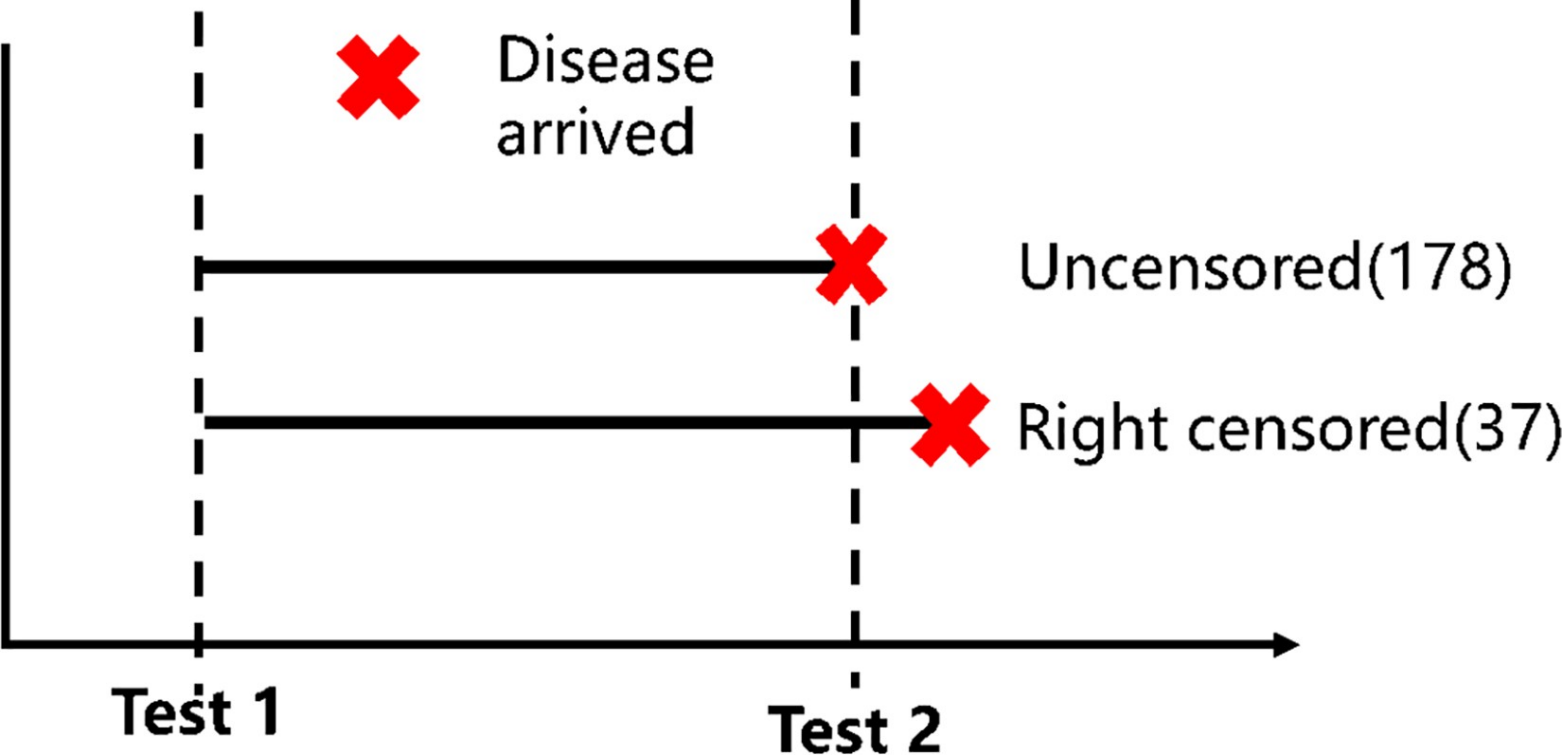
Censored data of COVID-19 arrival time.

To estimate the risk of disease arrival based on effective distance, we utilized the traditional Cox model and inverse distance model. Because our goal was to determine the arrival time of COVID-19 by airline at various countries, at first, we focused on the uncensored data, which included 178 countries that were affected as a result of air transportation. The right censored data included 29 countries with the IATA airports that had no COVID-19 infections. According to the definition of censored data above, disease spread began on Dec. 29, 2019 and ended on April 18, 2020 (Fig 6). The study period included the disease arrival time as well as observation time. The number of countries affected by COVID-19 during the observation time were considered to be uncensored data (178 countries), and the countries that were unaffected were right-censored data (29 countries), which might be impacted by COVID-19 in the future.

In the survival model, f(x) is the probability density function that an event will occur. S(t) is the survival function to determine the probability of survival (or that no event will occur) at time t. Eq 8 is the hazard function, or also the probability that an event will occur at time t.


$$S(t)=1-\int_{0}^{t}f(x)dx$$ (7)

$$h(t)=\frac{f(t)}{S(t)}$$ (8)

In Eqs 7 and 8, t is the time unit; S (t) is the probability that the event will not occur before time t; h (t) symbolizes the hazard function that the event will occur at time t or that no event will occur before time t. The simple hazard function ignores the heterogeneity of each individual element. The Cox model is used to analyze the effect of the explanatory variable multiplied by the baseline hazard function, as indicated below:

$$h(t)=h_{0}(t)\exp\left\{X_{i}\beta +\alpha \right\}$$ (9)

In Eq 9, h_0_(t) is the baseline hazard function; X_i_ is the explanatory variable (such as $D_{ij}^{SP},\;D_{ij}^{RW}$; β represents the coefficient and α is the intercept. The explanatory variable, in this case, can be either the effective distance or the geographical distance, which is calculated via the Euclidean distance formula ($D_{ij}^{Geo}=sqrt({\left({Lat}_{i}-{Lat}_{j}\right)}^{2}+{\left({Lon}_{i}-{Lon}_{j}\right)}^{2})$). Nah proposed a type of survival model based on effective distance as an “inverse distance” model (ID), which was used to estimate the spread risk of the Zika virus [13] and MERS [15]. The hazard of transmission was assumed to be an inverse of effective distance.


$$h_{j}^{SP}(t)=\frac{k}{D_{ij}^{SP}}$$ (10)

$$h_{j}^{RW}(t)=\frac{k}{D_{ij}^{RW}}$$ (11)

$$h_{j}^{Geo}(t)=\frac{k}{D_{ij}^{Geo}}$$ (12)

For the hazard function, as calculated by Eqs 10,11 and 12, Airport i is the disease seed and k is a constant parameter. In order to compare the Cox model to the ID model, the explanatory variable of the Cox model was subtracted from the distance log (log (Distance)). Eqs 13 and 14 show the two hazard functions of the Cox and ID models.


$$h_{j}^{Cox}(t)=h_{0}(t)\exp\left\{\log\left(D_{ij}\right)\beta +\alpha \right\}=h_{0}(t)\exp\left\{\alpha \right\}{\left[D_{ij}\right]}^{\beta }$$ (13)

$$h_{j}^{ID}(t)=\frac{k}{D_{ij}}=k{\left[D_{ij}\right]}^{-1}$$ (14)

In Eq 13, h_0_(t) is the exponential hazard function, which is a constant. The main difference between the two hazard functions is the order of distance. In addition, the density function *f*_*j*_(*t*), is based on the exponential distribution, and the integral of *f*_*j*_(*t*) is the cumulative probability of function F.


$$f_{j}(t)=h_{j}(t)\exp\left(-\int_{0}^{t}h_{j}(s)ds\right)$$ (15)

$$F_{j}(t)=1-\exp\left(-\int_{0}^{t}h_{j}(s)ds\right)$$ (16)

The density function f is used to calculate the probability that a disease has arrived in country j at an average time t, and the cumulative probability function F is the probability that the disease will arrive before time t. According to the parameter estimation of the traditional survival model, we used the likelihood function from Nah’s survival model [13], which included both uncensored and right censored data. The parameter, k, is estimated by maximizing the likelihood function. In Eq 17, t_j_ represents the arrival time of the disease at airport j, and t_m_ is the observation time (April 18, 2020). For example, the likelihood of uncensored data was calculated by analyzing 178 countries with the probability function *f*_*j*_, and right censored was determined via 37 countries which were not involved in Covid-19 importation with (1−*F*_*j*_(*t*_*m*_)), which represents the probability of an event occurring before the observed day (April 18, 2020, *t*_*m*_).


$$L(k)=\prod_{uncensored}f_{j}(t_{j})\prod_{right\;censored}\left(1-F_{j}(t_{m})\right)$$ (17)

## 3 Results

The following is a summary of our hypotheses.

1. Effective distance will have a significantly higher positive correlation with disease arrival time in various counties than with geographic distance.
2. The random walk effective distance will have a higher positive correlation with arrival time than the shortest path.
3. The effective distance can be included in the survival model to estimate the risk of spreading the disease to various countries.

First, we used the IATA flight schedule to establish an effective distance matrix among all the WAN airports. Table 3 shows an example of a partial effective distance matrix, and Table 4 displays the descriptive statistics of this matrix, which included 1,312 airports and 25,569 O-D links. The maximum distance was 13.573 kilometers (from the Amsterdam Schiphol Airport (AMS, Netherlands) to the Abruzzo Airport (PSR, Italy)), and the minimum was one. (These effective distances corresponded to 406 airports in 152 countries, which included 44 departure countries, 63 arrival countries, and 45 countries of both). As mentioned above, the effective distance was based on the passenger flow ratio. In other words, when the effective distance is equal to one, the airport would only link to one other airport and the percentage of passengers would be equal to one (1-ln (1) = 1–0 = 1). If there are no passenger flights between the two airports, their effective distance would be infinite due to the fact that there would be no travelers on those planes (1-ln (0) = 1- (-∞) = ∞). Therefore, these outliers were removed from the effective distance matrix (0 or infinity with which detection is infinite () function of R). For example, the effective distance from WUH to HND is infinite, because there are no direct flights between them. It must be noted that an infinite effective distance was not included in Table 3.

**Table 3:** An example of an effective distance matrix (an infinite effective distance).

Origin Airport Destination Airport	WUH	BKK	HND	ICN	IAD	TPE
WUH	-	3.21	Inf	3.64	Inf	4.09
BKK	6.30	-	5.09	4.12	Inf	4.62
HND	Inf	3.84	-	4.58	Inf	5.53
ICN	6.79	4.24	5.89	-	7.13	4.37
IAD	Inf	Inf	Inf	5.00	-	Inf
TPE	6.82	4.82	6.42	4.04	Inf	-

**Table 4:** Descriptive statistics of the distance from Wuhan airport.

	Min.	Max.	Median	Mean	Std.
Effective distance	1.000	13.573	5.570	5.542	1.809
Shortest path	3.209	18.862	12.818	12.512	2.975
Random walk	3.095	11.079	8.409	8.111	1.592

As previously mentioned, the shortest path and random walk are two types of disease spread path theories that require effective distance to establish the WAN. Because diseases do not always spread through a direct flight or the shortest path, effective distance can be calculated using the disease arrival probability function [10]. The descriptive statistics for the shortest -path and the random -walk effective distance from Wuhan Airport to airports throughout the world are shown in Table 4. It should be noted that we assigned only one airport for each country through which the disease could spread. There are 178 airports with effective distances from the Wuhan Airport (China). The minimum shortest-path and random-walk effective distances are 3.209 and 3.095 respectively (both are approximately three). However, the maximum values of the two types of effective distance were quite different (18.862 vs. 11.079). According to previous study results [10, 18], use of the random-walk effective distance method (which accounts for multiple possible paths) tends to lead to an underestimation of arrival time more frequently than the shortest path method (which includes only one path). Furthermore, the median and mean also indicated a similar trend, which is consistent with previous studies.

**Fig 7:**
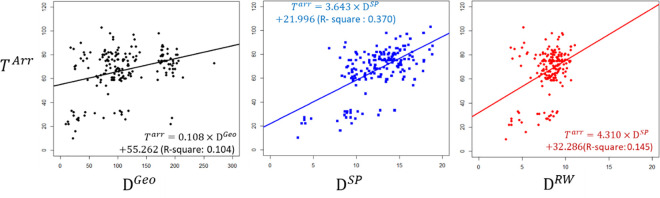
Scatter plot of the geographic distance. (a) the shortest-path and the random–walk effective distance (c) from Wuhan Airport to the other airports, and the arrival time of COVID-19.

A scatter plot was used to illustrate how differences in arrival time are correlated with the effective distances of the shortest path, random walk or geographical distance. In Fig 7, the x-axis illustrates the geographical and effective distance from Wuhan to other airports that have been associated with importation. The y-axis shows the actual arrival time based on online searches of confirmed cases. (Fig 7A–7C) show regression models of geographical, shortest-path, and random-walk effective distance. It is clear that the linear models of each distance all have significant coefficients and interceptions. The effective distance of the shortest path coefficient in the regression equation was 3.643, which represents the mean increase of COVID-19 arrival time in days for every additional one unit of effective distance. The intercept may represent the difference between the report time of the first confirmed case and the actual time it took for the first person to become infected.

As mentioned above, researchers have found that the multiple paths (e.g. random walk) technique, which is more realistic, is the most accurate method for estimating the arrival time of disease spread. Moreover, as previously stated, use of the shortest-path effective distance technique tends to lead to an overestimation of arrival time [10, 18]. We utilized a linear regression model (ordinary least square) to calculate the correlation of various distances with the actual arrival times. As seen in Fig 6, the R square of the random walk effective distance was lower than that of the shortest path, results that were inconsistent with previous studies. According to the scatter plot, this is due to more outliers being associated with the random walk effective distance than the shortest path. Also, the effective distance model performance was more accurate than that for geographical distance. The R square of the shortest path effective distance model was approximately 0.4, which indicates that this model can better explain the more realistic data patterns than those of the other two distance methods. In general, the shortest-path effective distance model yielded the best performance in this study.

The model fitting performance of the traditional Cox model vs. the inverse distance model, shown in Table 5, was based on two types of distance. The log likelihoods of the models were also comparable, meaning their performances were similar. To compare the traditional Cox model to the inverse distance-based model, we chose a log of shortest path effective distance as an explanatory variable of the Cox model. This was positive which indicates that countries with greater effective distance from Wuhan would be more likely to have longer disease arrival times. For the inverse distance-based model, k was the scale parameter of the hazard function (hazard function = k / effective distance), which is a constant that is used to predict when an event will occur. For example, a higher level of k is associated with a greater risk of COVID-19 and a shorter arrival time in all countries. The hazard functions (h (t)) of survival models are used to estimate the importation risk at time t, so the hazard at arrival time (h(T^arr^)) should be significant.

**Table 5:** Survival model results with the shortest path of effective distance.

	Cox model		Inverse Distance model	
			(Nah et al., 2016)	
	Effective distance	Geographic distance	Effective distance	Geographic distance
			($k/D_{ij}^{SP}$)	($k/D_{ij}^{Geo}$)
Intercept.	1.687 *	3.760 ***		
distance	1.090 ***	0.147.		
Parameter k			0.148	0.961
Log likelihood (model)	-960.2	-966.6	-954.9	-990.9

Significant Code: 0.0001’***’; 0.001’**’; 0.01’*’; 0.05 ‘.’; 0.1 ‘ ‘

As seen in Table 5, the model results showed that the log likelihood of the effective distance survival models was higher than those of the geographical distance model. Thus, probability distribution of disease arrival time can be calculated based on these parameter estimates. Although the two models had similar log likelihoods, their importation risk estimations were slightly different. For more information about this finding, Table 6 provides a summary of the various probability estimations of actual arrival times.

**Table 6:** Descriptive statistics of the arrival probability based on the Cox model and the effective distance model.

	Min.	Max.	Median	Mean	Std.
ID (Eff)	0.253	0.840	0.559	0.547	0.096
ID (Geo)	0.093	1.000	0.476	0.512	0.217
Cox (Eff)	0.260	0.850	0.555	0.545	0.098
Cox (Geo)	0.135	0.730	0.550	0.536	0.108

**Fig 8:**
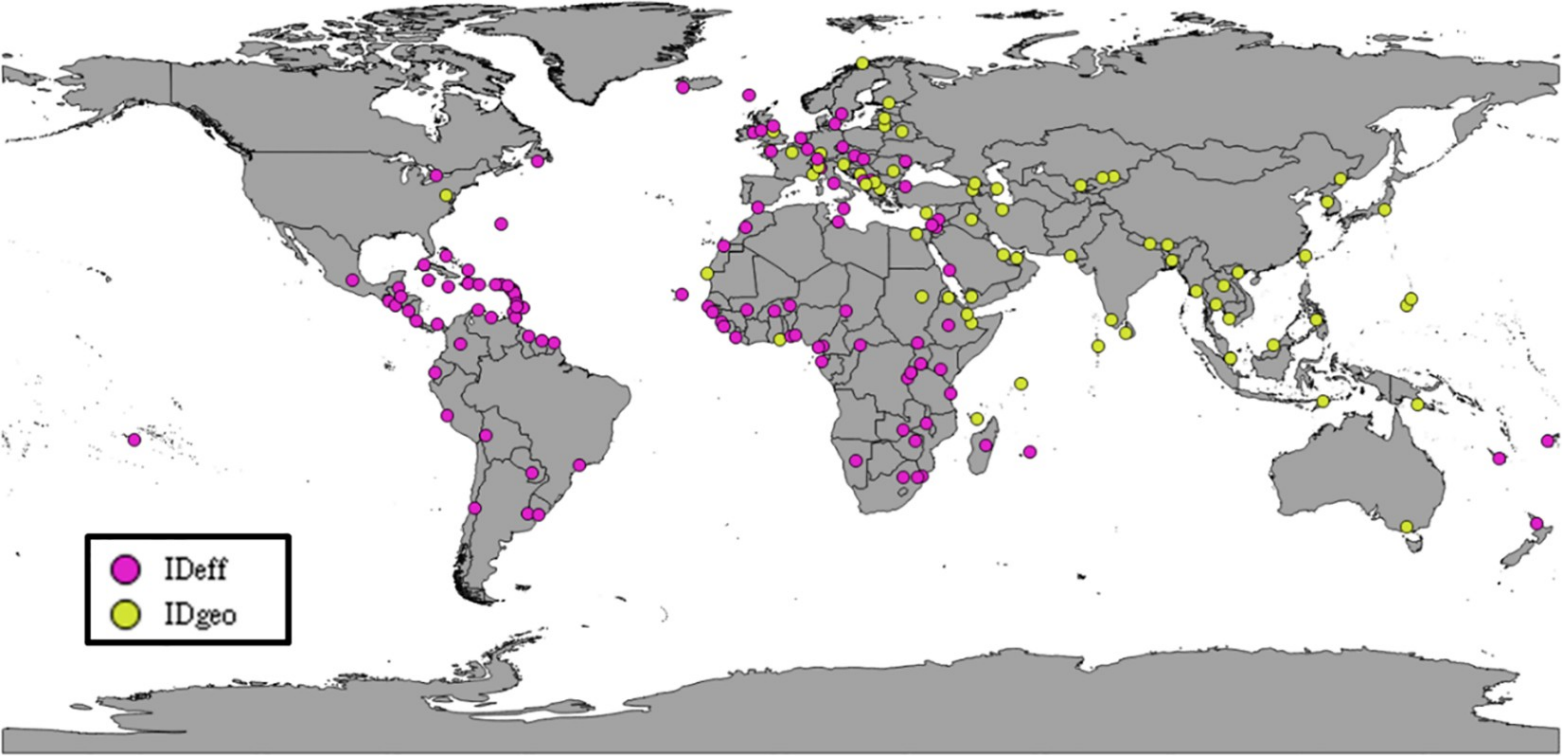
The map of maximum risk.

As shown in Table 6, the mean importation risk estimation with effective distance was greater than that of the geographical distance. Also, the standard deviation of importation risk estimation of geographical distance was larger than that of effective distance. The ID (Eff) model had the highest mean of importation risk and its log likelihood was also the highest, which shows that it yielded the best performance. Fig 8 shows the model that can provide the most accurate risk estimation for each country. The purple points are the importation airports which had the highest risk estimation according to the ID (Eff) model. The yellow points represent the countries with highest risk according to the ID (Geo) model.

There are 112 importation countries of COVID-19. As shown in Fig 8, approximately 63% have a higher risk estimation according to the ID (Eff) model, while the other countries with a higher risk of ID (Geo) are closer to the Wuhan Airport. This result indicates that the countries nearest to China are inconsistent with this hypothesis, because the geographical distance yielded a higher importation risk.

## 4 Conclusions

The goal of this study was to use the effective distance technique to establish a worldwide airport network in order to estimate disease spread. Two types of effective distance (shortest path and random walk) based on historical airline data (2017 IATA) and airport connection links were used to compare the predictability of the date of disease arrival. The conventional COX survival model and inverse distance model including effective distance were used to estimate the importation risk of disease.

After analysis, the results yielded several interesting findings.

- Effective distance performed better than geographical distance.

According to our results, effective distance provided better predictive performance than geographical distance, with the results confirming our expectations based on R-square. This finding is also consistent with Brockmann’s findings [11]. Although the IATA flight data of 2017 may not reflect the current reduction in air traffic due to the impacts of COVID-19, the metric of airline passenger flow is still more effective for estimating the arrival time of disease than geographical distance. For example, although Mactan–Cebu International Airport (IATA: CEB) in the Philippines is geographically closer to Wuhan Airport than the Paris Charles de Gaulle Airport (IATA: CDG) in France, according to the disease arrival time data utilized in this study, the later was affected by COVID-19 before the Philippines, which is consistent with this actual disease path.

- Random-walk effective distance did not perform better than shortest-path effective distance.

We found the model performance of the random-walk effective distance to be inferior to that of the shortest-path effective distance. This result is inconsistent with previous findings [18]. As previously mentioned, the random walk technique was developed to overcome the limitations of the simple path propagation by considering all possible paths within the airport network. A possible reason for this poor predictive power is that we used out-of-date pre-Covid IATA flight data that did not take into account the decreased number of flights due to COVID-19. Adiga et.al 2020 included a more recent IATA passenger flow dataset from Feb, 2020, which yielded higher model performance for the shortest path (R-square: 0.78) than our study (0.39) [16]. Unlike our arrival time dataset, their study included an analysis of the countries that were affected by COVID-19 before Feb 24. Thus, their results may not have been impacted by the multiple disease seeds which resulted in better model performance.

- The inverse distance survival model performed better than the Cox model.

We compared two survival models: the traditional cox model and the inverse distance survival model [13]. With regard to the uncensored data, the ID (Eff) model generated the highest importation probability at the actual arrival time. When we compared the ID model of effective distance to the geographical distance, we found that the arrival times in 66 uncensored countries (approximately 37%) were inconsistent with our original hypothesis, and most of them had higher risk estimation according to ID (Eff). This comparison will help scholars to better understand the characteristics of these survival models and to develop more accurate applications in the future. Due to the fact that risk assessment of former models was based only on the effective distance, these models can only be used to determine the importation risk of disease via air transportation.

The key results are listed below:

1. The effective distance model was found to more accurately predict the arrival dates of COVID-19 than the geographical distance model.
2. However, if out-of-date, pre-Covid airline data is included, the path of disease spread might not follow the random walk theory due to the recent flight suspensions and travel restrictions during the epidemic.
3. Lastly, with regard to effective distance, the inverse distance survival model and the Cox model yielded very similar importation risk estimates. Therefore, the possible application of this study is that if a new pandemic outbreak occurs in the near future, we can use out-off date airline flow to estimate the effective distance and then use the shortest path strategy to build the survival model.

There are several limitations of the current study, the first of which is the input data time scale. Because effective distance is designed to calculate typical percentages of passengers at all airports, this monthly or yearly flight data cannot capture the actual time the passengers spend traveling between airports. In addition, historical flight data was used in this study to establish effective distance. Thus, our model was unable to accurately represent the current traffic flow that is associated with disease spread. Some large airports with high traffic flow may became new disease seeds, which may influence the predictive performance of effective distance from the Wuhan Airport.

Future scholars may wish to collect more updated and detailed passenger flow data from additional airline databases. For example, not only does the OAG database include booking data for all flights, this platform can export passenger flow data each month. By using more current or updated airline datasets, researchers will be able to establish a more accurate global airport network which will help them to calculate effective distance more efficiently. Furthermore, this technique can be used to pinpoint the locations of multiple disease seeds. It will allow future scholars to reproduce the propagation path of COVID-19 using a dynamic numerical simulation model and to locate other possible disease seeds (such as in Italy and Iran) to prevent future peaks of this virus. In addition to the arrival time and the time of disease peak, cases of reinfection also require attention. Lastly, agent-based modeling is a common technique for simulating the status of individuals. In the future, researchers may wish to compare the stable epidemic model to the dynamic simulation model (agent-based model) in order to obtain a better understanding of the transmission process of COVID-19.
